# Parenting in place: Young children's living arrangement and migrants' sleep health in South Africa

**DOI:** 10.1002/psp.2692

**Published:** 2023-07-11

**Authors:** Sangeetha Madhavan, Seung Wan Kim, Michael White, Xavier Gomez‐Olive

**Affiliations:** ^1^ Departments of African American Studies and Sociology University of Maryland College Park Maryland USA; ^2^ Department of Sociology University of Maryland College Park Maryland USA; ^3^ Population Studies and Training Center Brown University Providence Rhode Island USA; ^4^ MRC/Wits Rural Public Health and Health Transitions Research Unit (Agincourt), School of Public Health, Faculty of Health Sciences University of the Witwatersrand Johannesburg South Africa

**Keywords:** childbearing, migration, parenting, sleep, South Africa

## Abstract

Migration research tends to treat childrearing as a secondary role for migrants. By prioritising the economic objectives of migration, most models present migrants as either delaying childbearing or, if they have young children, not living with them. However, migration has become increasingly feminised, the types of mobility more varied, while the returns to migration remain uncertain at best. At the same time, norms around childrearing are shifting, and the capacity of kin to take care of children may be weakening. In such contexts, migrants may not want to or be able to be separated from their children. Confronting such difficult decisions and their consequences may be reflected in poor sleep health for the migrant parent. We draw on data from the Migration and Health Follow‐Up Study (MHFUS) in South Africa to examine the following questions: (i) To what extent is children's coresidence associated with sleep health for migrant parents? (ii) Do effects vary by sex of migrant? and (iii) Do effects vary by location of migrant? Results from propensity score matching confirm that migrants who coreside with all their young children are more likely to experience healthy sleep compared to those who have nonresident or no young children. However, stratified analysis shows that these effects are only significant for women and those not living in Gauteng province. The value of these findings is underscored by the need for research on the well‐being of migrant parents who are negotiating multiple agendas in economically precarious and physically insecure destinations.

## 1 INTRODUCTION

The physical separation of children from their parents driven by internal labour migration has been a defining characteristic of Black family life in South Africa since the days of apartheid and through to the present. While high unemployment typically necessitates geographic mobility to secure a livelihood, conditions in the destination area are not secure enough to accommodate children. Therefore, children remain in distant, often rural, homesteads in the care of extended kin and, when possible, financially supported through remittances from migrant parents. Whereas there has been extensive research in many world regions, including South Africa, on the *children left behind* (Gaydosh, 2017; Mazzucato et al., 2015; Townsend et al., 2002), we know little about the relationship between children's coresidence and migrant parent well‐being. Whereas ‘stretched households’ (Spiegel et al., 1996) continue to be a common feature of Black families in South Africa 30 years after the collapse of Apartheid, there are two significant alterations: migration has become increasingly feminised and the role of extended kin in caring for children is no longer assured. In this analysis, we centre parenting within the internal migration process and focus on sleep health by asking the following questions: (i) To what extent is coresidence with young children associated with sleep health for migrant parents? (ii) Do effects vary by sex of migrant? and (iii) Do effects vary by destination? To address these questions, we leverage data from the Migration and Health Follow‐Up Study (MHFUS) in South Africa.

The importance of these questions is underscored by four critical issues. First, given the centrality of mobility in the lives of many in the Global South, we need new approaches and questions to understand how migrants manage the dual agendas of mobility for economic gain and parenting to fulfil personal and cultural expectations. Second, we know that the motivations to migrate vary by gender (Bonjour & Cleton, 2021). Given the increasing number of female migrants in contexts across the Global South (Statistics South Africa, 2020), it is critical that we better understand the extent to which the implications of mobility are gendered. Third, whereas research on the consequences of transnational migration on migrant well‐being is growing, the same cannot be said for internal migration despite the fact that the volume of internal migration (relocation within national borders) far outweighs the volume of international migration (Bell & Charles‐Edwards, 2013). Indeed, such research will advance our understanding of internal, that is, longer‐distance but intra‐national, migration as a process distinct from international migration in terms of how childbearing and parenting are managed. Fourth, while migration and mobility processes are institutionalised in many contexts, the need to consider the mental health of those involved is underscored by the considerable stress migrants endure from challenging economic conditions, suboptimal living environment and family pressures. We, therefore, have a unique opportunity to contribute to the growing research on adult mental health in the Global South (GBD, 2019) through an examination of migrant outcomes. Lastly, there is growing evidence for the critical role of sleep in ensuring good health outcomes via physiological and behavioural pathways (Colten & Altevogt, 2006; Hale et al., 2020) but the lion's share of this work comes from Western contexts. Our work advances our understanding of the relationship between sociodemographic processes and sleep health in a particular non‐Western context. In addition, we show the value of using detailed self‐reported sleep information in lieu of resource intensive biomarker data. The article begins with an overview of migration, gender and separation of families followed by a conceptual framework rooted in family‐based migration and parenting stress. We then present some context on South Africa followed by description of data, methods and findings. We close with a discussion of the implications of this work for both migration scholarship as well as policy.

## 2 BACKGROUND

Labour migration has been and continues to be a key livelihood strategy in many countries in the Global South and, in particular, sub‐Saharan Africa. In their analysis of DHS data from 31 countries, Cattaneo and Robinson (2020) found high rates of not just internal migration but repeated moves between rural and urban areas. A migration survey in five source regions of Ghana in 2013 and a follow‐up survey in 2015 showed that 65% of households had at least one migrant member in urban areas connected to rural family members through remittances (Awumbila et al., 2017). A recent study drawing on multiple demographic surveillance sites across the African continent shows annual rates of mobility ranging from 7% to 27% (Ginsburg et al., 2016). Historically dominated by men, migration has become increasingly feminised (International Organisation for Migration [IOM], 2019; Le Goff, 2016) as women seek better employment and educational opportunities similar to men. However, childbearing, particularly for women, continues to be valued (Dyer et al., 2008) and is sometimes seen as a way to secure a relationship (Swartz et al., 2018). Therefore, young women are likely to have multiple reasons to migrate.

When migrants are parents, the children often do not accompany them. Gaydosh (2015) found that migration is not only the most common cause of parental absence in Tanzania but is also quite lengthy. Improvements in transportation and communication have made it easier to maintain contact and visit (Peng & Wong, 2013; Ratha, 2011) though it is not clear the extent to which such improvements have actually strengthened relationships (Madhavan et al., 2017). The effects of separation can be examined from the perspective of both the children left behind and the migrant parents and entail costs and benefits. For children, some studies highlight the benefits for education mainly through remittances (Gaydosh, 2017; Townsend et al., 2002) while other work reports no effect on child mortality (Yabiku et al., 2012) or cognitive development (Nguyen, 2016). Yet other research suggests negative effects on psychological and emotional well‐being (Bennett et al., 2015a; Mazzucato et al., 2015). The relative cost and benefit of parental migration for children is likely to depend on a number of factors, including the amount and frequency of remittances, extent of physical contact between parents and children and attributes of the caregiver of the left‐behind children. Indeed (Yabiku et al., 2012) found that the best outcomes, in terms of child survival, were for children whose mothers reported successful migration by fathers (i.e., sending remittances).

How do such arrangements affect the migrant parents? Findings from the transnational families research suggests that migrant parents, particularly mothers, feel enormous guilt about leaving children behind and report feelings of loneliness and depression (Hondagneu‐Sotelo & Avila, 1997; Horton, 2009; Parrenas, 2001; Schmalzbauer, 2004). Haagsman et al. (2015), in their study of Angolan and Nigerian transnational parents in the Netherlands, found lower levels of subjective well‐being as did a more recent study also in the Netherlands of Nigerian parents (Berckmoes & Mazzucato, 2018). In one of the only such studies on internal migration, Cotton and Beguy (2021) draw on in‐depth interviews with migrant mothers separated from their children in a low‐income context in Nairobi to identify different strategies mothers use to maintain their connection to their children. They emphasise that most respondents found all of the strategies to be insufficient forms of mothering and expressed concern and significant stress about the well‐being of their children, even if they were in the care of close kin. Our analysis makes an important contribution to this scholarship by considering the effects of ***separation from*** as well as ***coresidence with*** children on migrant well‐being. Moreover, the increasing number of female migrants necessitates a gendered lens given that child rearing responsibilities continue to be borne by mothers.

### 2.1 Conceptual approach

We have long known that migration must be examined at the level of the family. Root and De Jong (1991) define the family‐migration system as links between family/kin at origin and destination. The move away from individual models of migration has been further solidified through the idea of ‘risk portfolio diversification’, a concept at the heart of New Economics of Labour Migration (Stark & Bloom, 1985). A critical component of NELM is ‘network and kinship capital’ to support migrant efforts in accessing labour market opportunities at destinations as well as taking on additional responsibilities in origin locations. By entering into voluntary contractual arrangements with migrants, nonmigrant kin may take care of young children of migrants with the expectation of receiving remittances from migrants. Extended kin have long played a critical role in supporting a socially dispersed model of childrearing (Caldwell & Caldwell, 1987; Madhavan et al., 2012; Parker & Short, 2009) sometimes institutionalised in child fosterage (Cotton et al., 2022; Isiugo‐Abanihe, 1985; Madhavan et al., 2018). At the same time, however, scholars have suggested that the extended family system is being overstretched and may not be able to adequately provide for the need of children out of parental care (Ariyo et al., 2019; Eloundou‐Enyegue & Shapiro, 2004) further exacerbating parental anxiety and stress. The migration literature, while centreing spatial mobility and the benefits of migration, is notably thin on this line of enquiry.

To address this gap, we draw from parenting stress theory to conceptualise the ways in which children's coresidence in the context of mobility affect migrant sleep health. According to Pearlin et al. (1981), parental role strain is rooted in social, economic and cultural factors but manifests in everyday life and can lead to negative mental health outcomes. Stress‐related to parenthood and parenting can manifest in sleep and depression. For example, women face considerable strain from parenting as a result of inadequate childcare options, difficult community conditions and cultural scripts around mothering expectations (McQuillan et al., 2019). This is particularly true for first‐time parents and, particularly, mothers (Levesque et al., 2020), in a qualitative study of new parents, found repeated complaints about poor sleep among the respondents. We also know that work‐related stress and balancing work‐family obligations (Kim et al., 2020; Mansyur et al., 2021) can manifest in sleep disturbance. Moreover, a comparative study of six countries including South Africa found that perceived neighbourhood safety is associated with better sleep (Hill et al., 2016).

For the study at hand, migrant parents face a host of challenges having young children live with them including insecure employment threatening financial provision, living in inadequate housing and exposure to high levels of crime, all of which would increase stress. At the same time, evidence from urban Kenya suggests that the ability and/or willingness of kin to provide financial and childcare support may have weakened (Clark et al., 2017). There is a growing literature on the social determinants of sleep (Hale et al., 2020; Kent et al., 2015) though almost all of it is from western contexts. Perceived strong social support is associated with good sleep (Nordin et al., 2008; Troxel et al., 2007) whereas strained relationships are linked to poor sleep quality (Brummett et al., 2006). Our focus on caregiving for children is an important contribution to this scholarship in that it speaks, indirectly, to the relationship between parents and other caregivers. This is further complicated by changes to parenting expectations across different historical periods (Elder, 2003). While having children raised by particular kin may have been normative and even desired at one time in much of Africa, socio‐cultural and economic changes have heralded new preferences for family organisation and parenting. Jackson's (2015) focus on matrifocality and modernity is particularly apt for South Africa where high rates of nonmarital childbearing combined with migration provide the ideal conditions for women living together in destination areas to pool limited resources and share child care. This might indeed more preferable than leaving children in the care of nonresident kin. Therefore, parental role strain can also come about when mothers perceive themselves as not meeting social expectations of the time and/or are more wary of kin support (Cotton & Beguy, 2021). In other words, female migrants may be facing increasing pressure to parent in place.

Given the multiple stressors linked to family and parenting obligations that migrants face, sleep health is an ideal choice for an outcome. Moreover, detailed self‐reported sleep information (as we use here) can provide such an indicator without recourse to highly resource‐intensive or clinical interventions, typically unavailable in low and moderate income settings. Specifically, we offer the following hypotheses:

Coresidence with young children is associated with poor sleep health for migrant parents because of stress from financial precarity and sub optimal living conditions

The effect on sleep will be more pronounced for women because of prevailing gender norms and gendered expectations.

The effect on sleep will be more pronounced in Gauteng, the most urbanised context, because of threats to safety

### 2.2 South African context

South Africa has a long history of labour migration put in place under apartheid when large numbers of Black African men migrated to cities to service the mines (Ramphele, 1993) and women moved to become domestic workers (Cock, 1989). Despite the collapse of apartheid, mobility has not abated but has become increasingly female (Camlin et al., 2014; Posel, 2004) with children and parents continuing to experience long periods of separation from one another. According to Statistics South Africa (2020), the number of migrant women in South Africa increased from 3.1% to 4.5% from 2012 to 2017. High unemployment necessitates mobility to urban areas to secure a livelihood but one that is not secure enough to accommodate children. Crime is a problem throughout South Africa but particularly so in cities and the townships surrounding them (Breetzke 2010, 2018;). Tellingly, in their review of rural‐urban migration in South Africa (Visagie & Turok, 2020) write, ‘South African cities remain inhospitable, relatively high‐cost environment for rural migrants’ (pp. 46).

The constraints of work environment and concerns for safety argue against the joint relocation of children with the migrant parent. Bennett et al. (2015b) found only 14% of children from origin households living in destination households and that children of migrant mothers, particularly if they are under the age of five, are more likely to move with them than with migrant fathers. They also found that children in the destination households of migrant fathers live in small, nuclear arrangements with their mothers whereas children of migrant mothers are in larger households often without fathers. More recent work has examined the experience of mother‐child coresidence with longitudinal data and found only 21% of migration events were classified as contemporaneous, joint mother‐child migrations; in other cases, the mother and child were not coresident before, after, or throughout the migration process (Posel, 2020). The decision to leave children in rural areas depends on the caregiver attributes. Older research has documented the negative effects of being raised by grandmothers on children's health due to their lack of knowledge about health (Cock et al., 1986). More recent work suggests that extended kin could play an important role in mitigating the effects of parental absence on children (Madhavan et al. 2012, 2017) but the effects on parents are unknown.

The continuing importance of migration as a livelihood strategy is made clear in light of the unemployment rate, which has remained above 20% for at least two decades (Statistics South Africa, 2011). Indeed, longitudinal research has provided strong evidence that rural‐urban migration is associated with declines in unemployment particularly for Black South Africans (Visagie & Turok, 2020). However, it is also true that employment options are limited and, often, unstable (Barchiesi, 2011). In the advent of apartheid's demise, the country has experienced significant shifts in gender roles with women (Dworkin et al., 2012; Morrell, 2002; Richter & Morrell, 2006)—and particularly Black women—attempting to leverage new rights and employment opportunities. Additionally, the country is undergoing a complex health transition in which both communicable, for example, HIV and noncommunicable, such as diabetes, diseases are present (Kahn, 2011; Marshall, 2004) and, in which, stress‐related health conditions and sleep are receiving increased attention (Buysse et al., 1989; Mai et al., 2019; Maume et al., 2018).

### 2.3 Site, data and methods

The research site is situated in Agincourt subdistrict of Mpumalanga Province, a rural area in northeastern South Africa. Agincourt is part of a former homeland, an area where Black South Africans were forcibly resettled between 1920 and 1970 under apartheid regulations. It is largely impoverished with limited employment opportunities, having adult unemployment rates often at or above about 30% (Collinson et al., 2016). Traditionally characterised as a sending area for men to work in the mining industry in Johannesburg, the pattern of mobility and the demographic composition of migrants has significantly changed. Labour migration has become more feminised and now involves moving to smaller towns and rural communities as destination areas. Female migration increased threefold between 1997 and 2001 (Collinson et al., 2016) and has continued to increase through 2017 (Statistics South Africa, 2020). Moreover, more women than men travel to destinations closer to Agincourt as it allows them greater flexibility to return home as needed (Collinson et al., 2016), particularly if they have children cared for by kin (Madhavan et al., 2012). The site is also home to the Agincourt Health and Demographic Surveillance System (AHDSS) which records all births, deaths, and in‐ and out‐migrations in a population of 116,000 covering 31 villages.

The AHDSS also provides an ideal platform to nest projects such as the Migrant Health Follow‐up Study (MHFUS). The 5‐year cohort study is aimed at better understanding the relationships between migration, urbanisation, and adult health in South Africa by following migrants who leave the AHDSS, usually to access employment in Gauteng province (which includes Johannesburg and Pretoria and perceived to be plagued by crime) or larger towns. The data collection began in 2017 with a target sample of 3800 individuals aged 18 to 39 drawn from the DSS. It included residents of the Agincourt site (nonmigrants) and those members who were not present but counted as part of the household. These ‘temporary migrants’ maintain contact with their origin households through remittances, phone and periodic visits. MHFUS collected detailed information on motivations for migration, residence histories, household rosters for both origin and destination households, remittances including relationship of receiver to migrant, and details about life in the destination location, employment, adult health and well‐being including a module on sleep quantity and quality. Full maternity and paternity histories were added to data collection in Wave 2. All respondents, regardless of migrant status or gender, were asked about all living children, including their age, sex and current location.

In Wave 1, 3103 interviews were completed comprised of 1886 (70%) nonmigrants residing in the Agincourt HDSS and 1221 (30%) migrants living outside of Agincourt. Out of the original sample of 3103, 92% (*N* = 3026) were successfully followed up in Wave 2 from which 2975 were successfully followed up by phone in Wave 3. For this analysis, we draw on the Wave 3 migrant sample of 1525 respondents. After removing those with missing values, our analytical sample is 1484. The dependent variable is sleep health, an index reflecting both sleep quantity and quality. The recommended hours of sleep is between 7 and 9 h; insufficient less than 7 h and oversleep is greater than 9 h (Hirshkowitz et al., 2015). While sleep deficiency is the more common sleep problem, oversleep can have negative implications for physical and mental health (Buxton & Marcelli, 2010). Moreover, other work has found oversleep to be much more prevalent among Black Africans in South Africa (Peltzer, 2017). To gauge sleep quality, we use the Pittsburgh Sleep Quality Index (PSQI), a 10‐item scale from 0 to 21 that uses a score of 6 or higher to categorise poor quality sleep. Bringing the two together, our dichotomous sleep health outcome is 1 (healthy) for those who have the recommended hours of sleep and a PSQI score < 6 and 0 (problematic) for those who experience oversleep (>9 h) or sleep deficiency (<7 h) or a PSQI ≥ 6. We also conduct sensitivity tests recoding oversleep as healthy.

We focus on children ≤age 5 as they are likely to be more dependent on parental connection. We first examine differences, along relevant descriptors. between migrants who have and do not have young children and, among those with young children, differences between those who live with all of them and those who do not. Following bivariate analyses of our variables of interest, we use logistic regression to examine the relationship between children's coresidence and migrant sleep health. We use a categorical exposure variable: live with all young children (ref), separated from at least one young child and have no young children. We also test for the effect of coresidence for just those migrants with young children. Control variables include a set of migrant parent characteristics: age, sex, partnering status (no partner, partner/spouse is in the current household, partner/spouse in the place of origin and elsewhere), employment status, last month's income (low, med, high), educational attainment, risk for depression measured by a CESD^1^ score ≥10, sleep health at previous wave and sending remittance. To confirm that the results are not driven by selection on sleep behaviour, we include sleep health in previous wave and do not find any differences in the results. We also control for contextual attributes, namely, migrant destination (Gauteng or non‐Gauteng), number of women of childbearing age in the household as a proxy for availability of child care and number of unemployed adults in the household to capture additional sources of stress. Because the CESD‐10 includes a sleep item, we conducted a sensitivity test removing the item and found no difference in the results. To examine the moderating effect of sex of migrant, we include interaction terms and also run stratified models to show within group differences. We do the same with destination but dichotomised as urban/rural to facilitate interpretation.

Because this is a cross‐sectional analysis, selection due to unobserved heterogeneity or omitted variable bias is highly likely. Might migrants who live with their young children differ from those who do not, beyond ways observed in a standard survey? While we address this through the inclusion of control variables in the regression models, it is a partial fix at best. We use propensity score matching (PSM) to more affirmatively discern the effect of parenthood and parenting on migrant sleep health through a quasi‐experimental approach for ensuring comparability between two groups (Littnerova et al., 2013). In our PSM analysis, we use ‘has at least one child living away OR has no young child’ as the ‘treatment’ with three different samples: (i) all; (ii) only female migrants and (iii) non‐Gauteng destination, in line with our three hypotheses. We match using the Nearest Neighbour specification on age, sex, employment status, and educational attainment.

## 3 RESULTS

Table 1 presents selected descriptives of the migrant sample stratified by parenthood and, for those with young children, stratified by children's coresidence.

**Table 1:** Sample descriptives by parenthood and children's living arrangements, Migration and Health Follow‐Up Study (MHFUS) Wave 3.

	All migrants % (SE)		Migrants with children ≤5% (SE)		
	Have a child ≤5	Have no children ≤5	All children coresident	At least one child nonresident	*N*
Mean age	31.0 (0.21)	30.7 (0.20)	31.5 (0.40)	30.7 (0.25)	1484
% Female	44.5% (2.1%)	40.5% (1.6%)	71.0% (3.4%)	31.8% (2.3%)	621
Partnering status					
Not partnered	65.4% (2.0%)	80.4% (1.3%)	42.0% (3.7%)	75.9% (2.2%)	1108
Partner coresident	21.9% (1.7%)	11.7% (1.1%)	49.4% (3.8%)	9.5% (1.5%)	231
Partner not resident	12.7% (1.4%)	8.0% (0.9%)	8.5% (2.1%)	14.6% (1.8%)	145
Highest educational attainment					
Prematric	26.0% (1.8%)	23.6% (1.4%)	24.4% (3.2%)	26.7% (2.2%)	364
Matric	51.0% (2.1%)	50.9% (1.7%)	46.0% (3.8%)	52.8% (2.5%)	754
Postmatric	22.8% (1.8%)	24.1% (1.4%)	29.5% (3.4%)	19.0% (2.0%)	347
Employment Status					
Formal	45.9% (2.1%)	43.5% (1.6%)	35.2% (3.6%)	50.8% (2.5%)	659
Informal	12.7% (1.4%)	14.4% (1.2%)	9.7% (2.2%)	14.1% (1.8%)	204
Not employed	41.3% (2.1%)	42.2% (1.6%)	55.1% (3.8%)	35.1% (2.4%)	621
Income					
Low	62.0% (2.0%)	64.6% (1.6%)	73.9% (3.3%)	56.7% (2.5%)	944
Medium	31.6% (2.0%)	28.1% (1.5%)	21.0% (3.1%)	36.4% (2.4%)	437
High	6.4% (1.0%)	7.3% (0.9%)	5.1% (1.7%)	6.9% (1.3%)	103
CES‐D score (mean)	2.6 (0.22)	3.7 (1.07)	3.1 (0.59)	2.4 (0.16)	1484
Sends remittance	33.4% (2.0%)	31.6% (1.5%)	22.7% (3.2%)	38.7% (2.5%)	481
*N*	566	918	176	390	

There are no notable differences by parenthood status on any attribute except partnering status and mean CESD score and partnering status. Migrants without any children under age 5 are more likely not to be partnered or living with the partner and also have a higher CESD score compared to migrants with a young child.

When we focus on living arrangements for those with young children, we find much more variation. Those who live with all their young children are much more likely to be female, living with their partners and, interestingly, have a higher proportion of ‘post‐matric’ in terms of educational level compared to those who have at least one young child living away. Employment status shows a distinct difference across categories. Migrants who have at least one young child living away are more likely to be formally or informally employed compared to those who live with all their children. Unemployment is much higher (55.1%) among those with all young children living with them, which would also explain the higher likelihood of being low‐income. The mean depression score is higher for those who live with all their young children (3.1) compared to those who have at least one child living away. Lastly, as expected, those who have at least one young child living away are more likely to send remittances (38.7%) compared to those who live with all their children (22.7%).

Table 2 provides figures for migrant destination and household attributes (at destination) by parenthood status and children's coresidence.

**Table 2:** Contextual attributes by parenthood and living arrangements, Migration and Health Follow‐Up Study (MHFUS) Wave 3.

	All migrants % (SE)		Migrants with children ≤5% (SE)		
	Have a child ≤5	Have no children ≤5	All children coresident	At least one child nonresident	*N*
Destination					
Gauteng	39.0% (2.1%)	46.2% (1.6%)	38.6% (3.7%)	39.2% (2.5%)	645
Other urban	30.7% (1.9%)	33.9% (1.6%)	22.7% (3.2%)	34.3% (2.4%)	485
Other rural	30.0% (1.9%)	19.6% (1.3%)	38.6% (3.7%)	26.2% (2.2%)	350
Household (at destination) attributes					
Number of women of reproductive age^a^					
0	72.6% (1.9%)	79.5% (1.3%)	42.6% (3.7%)	86.2% (1.8%)	
1	15.0% (1.5%)	11.8% (1.1%)	25.0% (3.3%)	10.5% (1.6%)	
1+	12.4% (1.4%)	8.7% (0.9%)	32.4% (3.5%)	3.3% (0.9%)	
Number of unemployed household members					
0	77.4% (1.8%)	82.7% (1.2%)	54.5% (3.8%)	87.7% (1.7%)	
1	16.4% (1.6%)	10.7% (1.0%)	30.7% (3.5%)	10.0% (1.5%)	
1+	6.2% (1.0%)	6.6% (0.8%)	14.8% (2.7%)	2.3% (0.8%)	
*N*	566	918	176	390	1484

^a^ Not including migrant parent.

Those migrants who have a young child are less likely to live in highly urbanised Gauteng (38.8%) and more likely to live in rural communities (30.7%) compared to those migrants without young children (46.2% Gauteng and 19.6% rural). Perhaps not surprisingly, those parents with a young child are more likely to have 1+ women of reproductive age in the household (not including the migrant parent) compared to those without young children. When we examine migrant parents with young children, those who live with all their young children are most likely to live in a rural community though are no less likely than those who do not live with all their young children to live in Gauteng. Household attributes also show variation across groups. The overwhelming majority of migrant parents with at least one child living away report having no woman of reproductive age in the household whereas those with all their children coresident are more likely to have more than one woman of reproductive age living in the household. However, it is surprising that 42.6% report having no women in this age group coresident. It is notable that migrant parents who have all their children living with them face a higher dependency burden in terms of unemployment compared to those who have at least one child living away. This suggests that additional childcare responsibilities may make it difficult to maintain employment.

Table 3 shows sleep quantity, quality and the sleep health index by parenthood and children's coresidence.

**Table 3:** Sleep outcomes by parenthood and children's living arrangements, Migration and Health Follow‐Up Study (MHFUS) Wave 3.

	All migrants % (SE)		Migrants with children ≤5% (SE)		
	Have a child ≤5	Have no children ≤5	All children coresident	At least one child nonresident	*N*
Sleep hours					
Recommended	53.4% (2.1%)	50.1% (1.7%)	57.4% (3.7%)	51.5% (2.5%)	650
Insufficient	19.4% (1.7%)	19.8% (1.3%)	11.9% (2.5%)	22.8% (2.1%)	292
Oversleep	27.2% (1.9%)	30.1% (1.5%)	30.7% (3.5%)	25.6% (2.2%)	430
PSQI > = 6	4.0% (0.9%)	5.2% (0.7%)	4.1% (1.5%)	4.5% (1.1%)	70
Sleep health index					
Healthy	52.5% (2.1%)	48.7% (1.7%)	56.3% (3.8%)	50.8% (2.5%)	744
Problematic	47.5% (2.1%)	51.3% (1.7%)	43.8% (3.8%)	49.2% (2.5%)	740
*N*	566	918	176	390	1484

*Note*: Recommended sleep hours: 7–9; Healthy PSQI < 6.

For the most part, those with and without young children are fairly similar in terms of sleep hours, quality and the sleep health index (confirmed by formal tests of significance). Such first‐blush comparisons call into question some of the prevailing expectations about migration, parent‐child coresidence, and stress (via asleep indicator), as embedded within the first hypothesis, but these results also argue for more comprehensive evaluation of the relationships.

More than 50% of the sample are classified as having recommended hours of sleep and very few have poor quality sleep as measured by a PSQI ≥ 6. When we compare living arrangements of children, we find that migrant parents with at least one young child living away report having insufficient sleep (22.8%) compared to those who live with all their children (11.9%). In terms of the sleep health index, those with all their children living with them are more likely to experience healthy sleep compared to those who have at least one young child living away. However, none of these differences are significant at the bivariate level.

**Figure 1:**
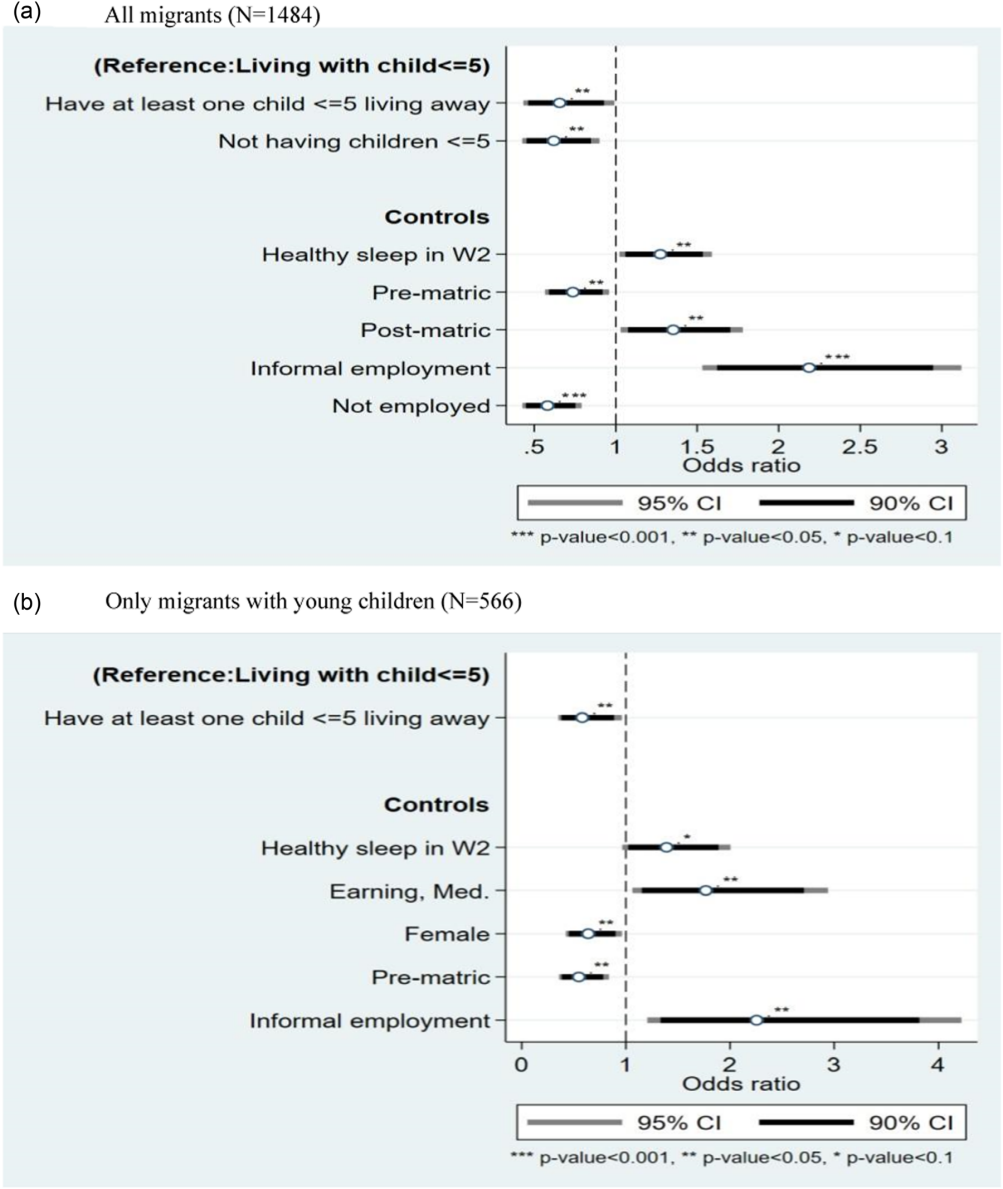
Logistic regression results predicting healthy sleep as a function of children's living arrangements, Migration and Health Follow‐Up Study (MHFUS) Wave 3 (a). All migrants (*N* = 1484), (b). Only migrants with young children (*N* = 566) Reference categories for control variables: Completed matric (secondary education); formal employment.

We now move to multivariate models to examine whether differences hold net of individual and contextual factors. Figure 1 shows the results of a logistic model predicting the odds of experiencing healthy sleep as a function of children's coresidence. Figure 1a includes all migrants with the reference category as ‘having all young children coresident’ while Figure 1b is restricted to only migrants with young children while employing the same reference category. In the interest of clarity, only significant effects are shown but all controls are included in all models.

Hypothesis 1 concerned coresidence and sleep itself. When we include all migrants (Figure 1a), we find that migrants who do not have any young children or have at least one child living away face lower odds of experiencing healthy sleep compared to migrants who coreside with all their young children net of having healthy sleep in the previous wave. For education, we find that those who did not complete matric face lower odds of having healthy sleep compared to those who completed matric where those who went beyond enjoy better sleep. In terms of employment, we find that those with informal employment are more than twice as likely to experience healthy sleep compared to those with formal employment whereas the unemployed face lower odds of having healthy sleep. Taken together, these findings suggest that the lack of any effects at the bivariate level (Table 3) is attributable to omitted variables with effects that mask the effect of children's coresidence. To ensure that these findings are not a reflection of time substitution—those without young children or are unemployed have more time to ‘oversleep’—we ran the model with oversleep categorised as healthy sleep in the dependent variable. The results did not change.

When we restrict the sample to only migrants with young children (Figure 1b), the effect of children's coresidence remains the same. Migrant parents who have at least one young child living away from them face *lower odds* of experiencing healthy sleep compared to those who have all their young children living with them. Models including sleep health in previous wave (not shown) did not change the results. This result contradicts the expectation that coresidence with young children is detrimental for sleep due to parenting stress. While the effect of informal employment remains positive and significant, unemployment becomes nonsignificant. To probe the employment effects further, we ran a cross tab of employment type and sleep health and found no significant differences. One possible explanation can be found in the consumer choice model (Szalontai, 2006) that suggests that those who earn higher hourly wages experience poorer sleep because the price of sleeping is higher.

**Figure 2:**
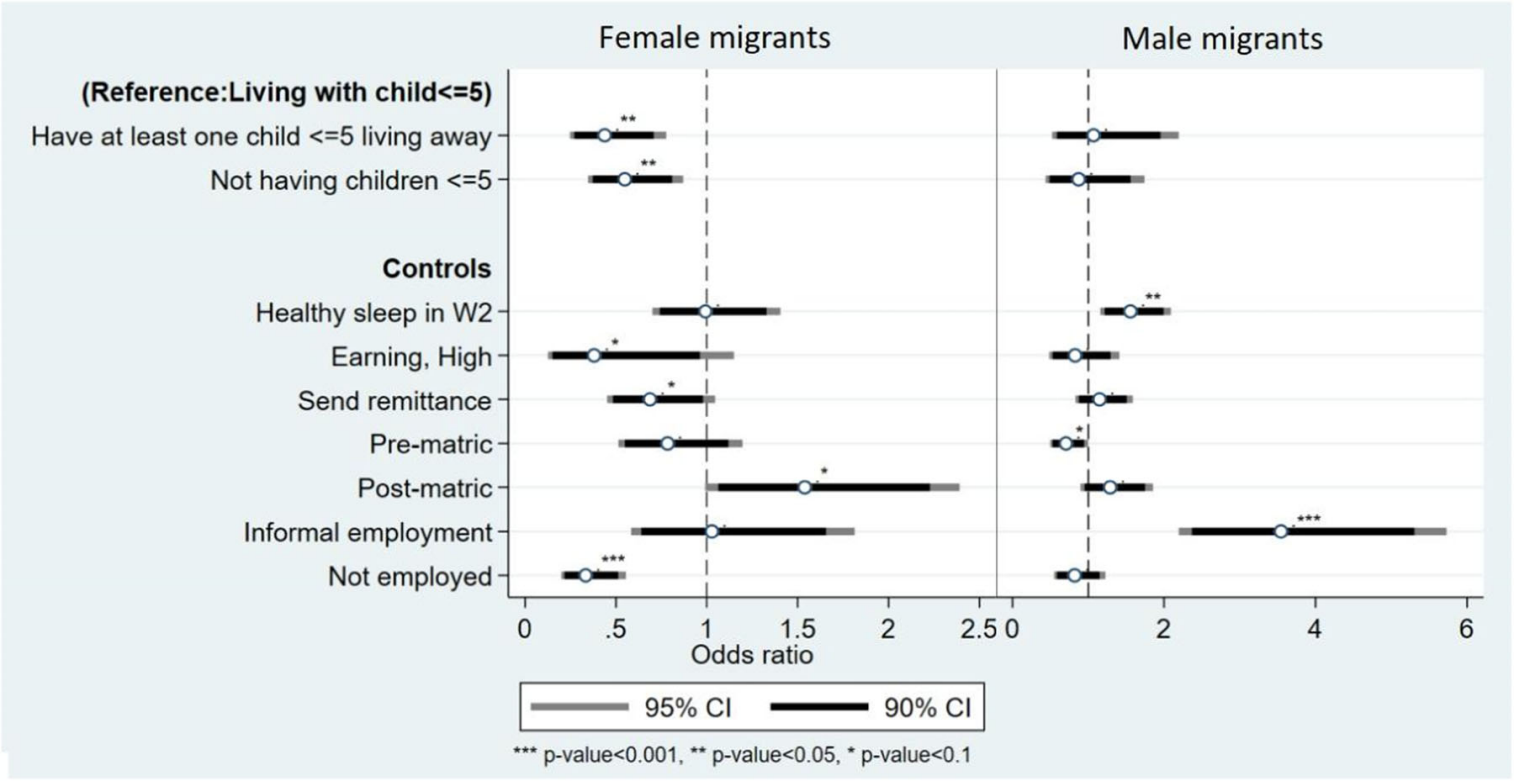
Logistic regression results predicting healthy sleep as a function of living arrangements stratified by sex of migrant, Migration and Health Follow‐Up Study (MHFUS) Wave 3 (all migrants) reference categories for control variables: no earning or low income (<R3,200 in the last month); completed matric (secondary education); formal employment.

Hypothesis 2 stated that the effects on sleep depend on the sex of migrant parent. The model including an interaction term for children's coresidence and sex of migrant showed no significance (not shown). Therefore, the second hypothesis that effects are more pronounced for women is not supported. However, there are interesting differences *within* sex as shown in the sex‐stratified models in Figure 2.

We find that, whereas women with at least one young child nonresident and women without any young children face significantly *lower odds* of experiencing healthy sleep compared to those with all young children coresident, no such effect is evident for men. Interestingly, having high‐income earnings also has a negative effect, albeit weak, on sleep health for women. Lastly, it is notable that women who send remittance face worse sleep than those who do not even after controlling for employment status. These findings suggest that women experience stress generated from not meeting cultural and/or relationship expectations of parenting as well as fulfilling kinship obligations in ways that men do not. Such results suggest a gender differential in how the migration‐coresidence‐stress process may operate.

**Figure 3:**
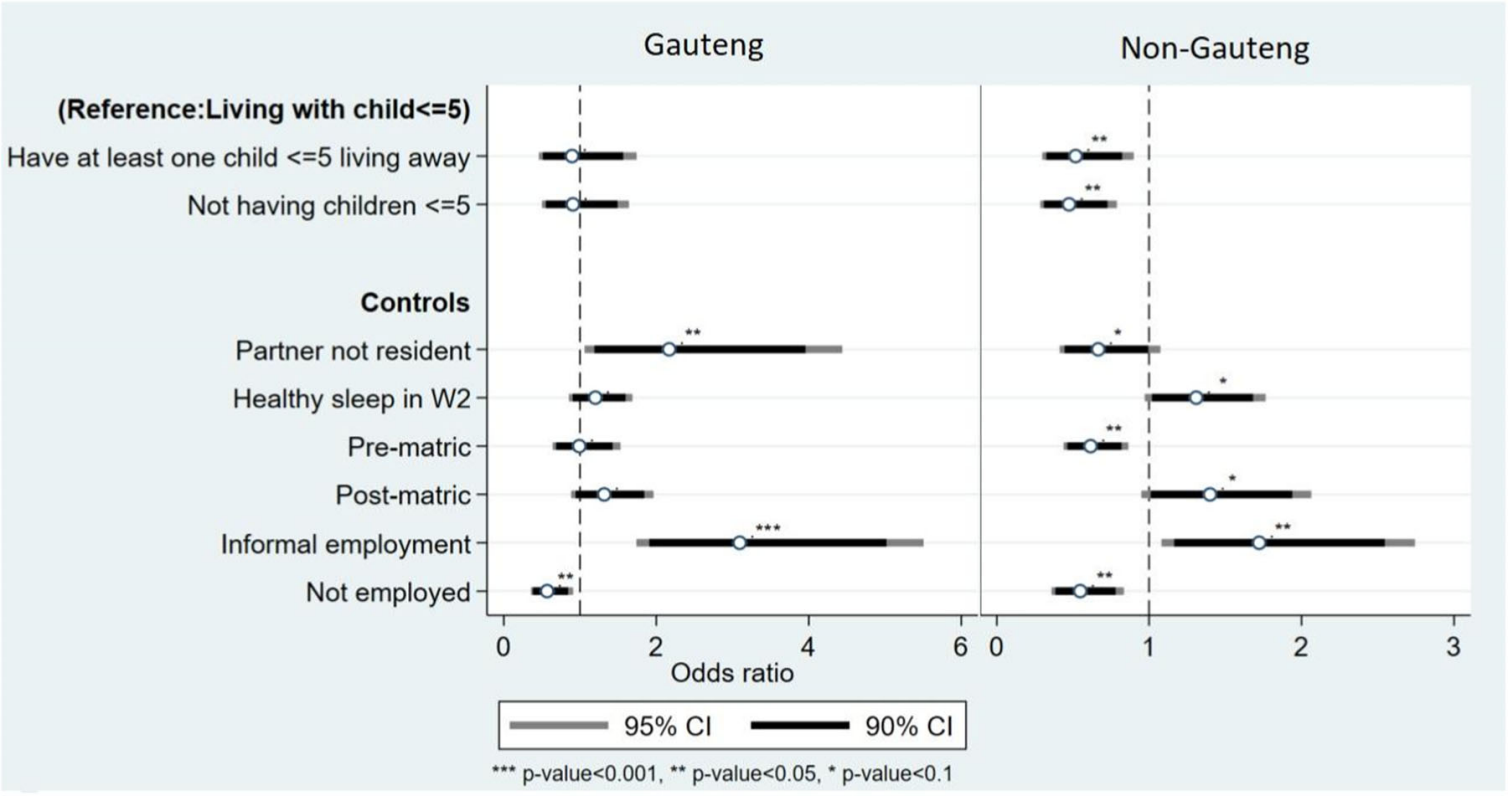
Logistic regression results predicting healthy sleep as a function of living arrangements stratified by residence of migrant, Migration and Health Follow‐Up Study (MHFUS) Wave 3 (all migrants) reference categories for control variables: Not in a union, completed matric (secondary education); formal employment.

Hypothesis 3 concerned whether the effects on sleep depend on the destination of migrant parent. The model including an interaction terms for children's coresidence and location of migrant showed no significance (not shown). Therefore, the third hypothesis that effects are more pronounced for migrants in Gauteng, the most urbanised location, is not supported. However, there are interesting differences *within* destination as shown in the stratified models in Figure 3.

Children's residence only appears to matter for migrants who live outside of Gauteng. Those who do not have any young children or have a young child living away face lower odds of healthy sleep compared to those who live with all their young children. This may be reflective of the overwhelming number of stressors present in Gauteng, which may, in turn, mute any significant effects of children's coresidence. We also find that having nonresident partners (compared to being partnered) improves sleep health in Gauteng but has the opposite effect for locations outside of Gauteng.

Table 4 presents results of the propensity score analysis to more precisely account for selection bias.

**Table 4:** Results of propensity score analysis, Migration and Health Follow‐Up Study (MHFUS) Wave 3.

Treatment	Average treatment effect	*p*‐value	Average treatment effect on the treated	*p*‐value	*N*
(All) Have no children ≤5 OR at have least one child living away versus Living with all children ≤5	−0.15 (15%)	0.005**	−0.14 (14%)	0.009**	1484
(Only females) Have no children ≤5 OR have at least one child living away versus Living with all children ≤5	−0.14 (14%)	0.026*	−0.12 (12%)	0.07^+^	621
(Only non‐Gauteng) Have no children ≤5 OR have at least one child living away versus Living with all children ≤5	−0.23 (23%)	0.001**	−0.23(23%)	0.002**	839

*Note*: Nearest neighbour matching based on respondent sex, age, education (matric/postmatric vs. other), and employment (formal, informal, and unemployment).

***.001; **.01; *.05; ^+^.10.

The average treatment effect on the treated (ATET) shows that the treatment increases the odds of healthy sleep in the first model (have a child vs. not among all) and lowers the odds of healthy sleep in the second model (children living away vs. coresident among female migrants) and third model (children living away vs. coresident among non‐Gauteng only). Where the p‐level is significant, we can have some confidence that the effect is not entirely due to selection on sex, age, education and employment. The first model, the treatment results in a 14% decrease and is highly significant, giving us more confidence that the effect of living arrangements on sleep cannot be explained by selection. When we limit to just female migrants, we find a marginally significant decrease in the odds of experiencing healthy sleep. When we focus on migrants who do not live in Gauteng, we find that those who have no young children or have a young child living away are 14% less likely to have healthy sleep. In sum, while we cannot rule out selection as a contributing factor in explaining the results and recognise that the PSM only includes a limited set of covariates, the results are suggestive of a possible causal pathway between children's coresidence and sleep health.

## 4 DISCUSSION

In this paper, we set out to investigate an understudied dimension of family‐based migration in the Global South—the relationship between children's residential arrangements and migrant health and well‐being. Further, our analysis has advanced the knowledge base on adult health in non‐Western contexts and, in particular, the social determinants of adult sleep health. Drawing on a unique data set covering both migration patterns and health outcomes in South Africa, we found that migrants who coreside with their young children are *more likely* to experience healthy sleep compared to those with nonresident children as well as those without any young children. Second, while the effect of children's coresidence on sleep is not more pronounced for women as we expected, women who parent in place reap some sleep benefits compared to their peers whose children live away from them. Third, stratified analysis shows that these effects are only significant for those who live outside of Gauteng, one of the most urbanised areas of South Africa. Results from propensity score matching confirm that selection on key attributes, namely, age, sex, employment status, and educational attainment, are not driving the results.

To situate these findings in the broader scholarship, we revert to our initial hypotheses. The literature on parenting and stress points to a negative effect of parenting on sleep quantity and quality. Moreover, it has also been shown that post traumatic stress disorder is associated with poor sleep (Lipinska et al., 2017; van Wyk et al., 2016). Migrants are likely to have high levels of chronic stress through exposure to multiple challenges. Therefore, one would expect (H1) that having coresident children would only heighten stress manifesting in poor sleep (Lévesque et al., 2020). Our results suggest quite the contrary. While there is little doubt that migrant parents who live with their children most certainly endure considerable stress, our analysis suggests that those who do not have their children live with them may face yet another stressor from ceding control over their children's welfare coupled with burdening kin with additional responsibilities. Our second hypothesis (H2) was aimed at understanding the connection between migration and childrearing as a gendered process given the increase in the number of female migrants—both internal and international. The marginal significance shown in the PSM suggests that migrant women who parent in place are likely selected to handle stress more effectively than those with nonresident children. Last, we did not find that being in Gauteng (H3)—arguably the most physically and economically insecure context—moderated the effect of having coresident children. However we did find a positive effect of coresident children on sleep for migrants *living outside of Gauteng*. While there is no clear explanation for this finding, it underscores the need to redouble research on internal migration processes at a time when the opportunities—both actual and perceived ‐ associated with place are constantly shifting and likely to affect how decisions about who, when and for how long people move.

The interpretation of our results should consider several limitations. First, sleep quality and quantity are self‐reported. Indeed, findings from other work in the same field site (Gómez‐Olivé et al., 2018) has shown that self‐reported measures tend to exaggerate sleep quality. Related to this is the practice of mothers cosleeping with young children, a common phenomenon in cramped, township housing in South Africa. While some research has shown a link between cosleeping and poor sleep quality (Volkovitch et al., 2015), the MHFUS respondents may report good sleep because they perceive they are protecting their children in the context of unsafe environments. Second, despite our best efforts to address selection through propensity score matching, the lack of any timing data make it difficult to establish lines of causality with certainty. Third, while we control for depression in the models, the bidirectional relationship of sleep and depression necessitate future work to unpack the effects through structural equation modelling. Fourth, our focus on young children limits the generalisability of the findings to the larger question of child rearing and hope that future work will include older children. Lastly, we limited this analysis to the migrants in the MHFUS study whereby not discerning whether sleep effects are a function of mobility or separation of parents and children which can occur outside the context of migration. Also missing is data on frequency of contact in person or through phone and social media, both ubiquitous in South Africa, and likely to moderate the effects of living arrangements on sleep.

Despite these limitations, this analysis makes a worthy contribution to the growing body of research on migration and health through the lens of parenting. Our approach and analysis seek to widen the scope of migration studies. We concur with De Haas (2010) that there is much in common between internal and international migration strategies, and that the concept of livelihood strategy has points of intersection with New Economics of Labour Migration (NELM) framework. However, our analysis complicates standard portrayals of a family based migration strategy in which the returns to migration are maximised through delaying or temporarily stopping childbearing or enlisting extended kin to provide child care. Accordingly, we aim to bring a deeper understanding of intrafamilial (and household) dynamics into these strategies, especially incorporating the patterns of parenting that obtain in low‐ and middle‐income (LMIC) environments. Additionally, it underscores the need to conduct more research on migrant stress as a function of economic success or failure, assimilation to new living conditions, connections to kin and, quite importantly, anxieties around childbearing and the ability to parent in place. Since true experiments are unlikely to be conducted to manipulate family configurations and coresidence, carefully implemented studies with high‐quality observational data are likely to prove invaluable. Future research should also consider other factors, for example, sleep health, to fully appreciate the costs of migration for men and women in contexts marked by economic precarity and shifting norms around gender roles and family obligations. We trust that such richer data and melded frameworks can help improve our understanding of many patterns of migration, whether internal or international, and always set in the context of something larger than an individual pecuniary decision.

## CONFLICT OF INTEREST STATEMENT

The authors declare no conflict of interest.

## Data Availability

The data are available upon request.

## Footnotes

1 CESD refers to the Center for Epidemiological Studies Depression scale.
